# The effect of minocycline on the gut microbiome in individuals with alcohol use disorder and healthy controls

**DOI:** 10.64898/2026.10.09.758026

**Published:** 2026-10-16

**Authors:** Carolyn M. Doty, Daniel J. Brady, Jennifer J. Barb, Summer L. Thompson, Natalia Rincon, Brittney Browning, Deanna L. Kelly, Melanie E. Bennett, Greg Elmer, Lorenzo Leggio, Brian J. Brandler, Daniel J.O. Roche

## Abstract

Alcohol Use Disorder (AUD) is associated with altered gut microbial composition. Yet, many microbiome studies have included AUD populations with co-occurring conditions (e.g., alcohol associated liver disease; ALD), limiting insight into unique contributions of AUD to gut dysbiosis. Further, preclinical studies show that gut microbiome composition can mediate the behavioral effects of minocycline, including alcohol-related and affective behaviors, but no studies have tested these effects in humans with AUD. This study aimed to 1) compare gut microbiome features between healthy controls (HCs) and individuals with AUD, without ALD, and 2) determine the effects of minocycline on the human gut microbiome. We conducted a four-week randomized, double-blind, placebo-controlled trial of minocycline (100-200 mg/day) in adults with AUD (n=31) and HCs (n=26). We collected fecal samples (n=244) at baseline and each weekly visit for 16S rRNA gene sequencing. Alpha diversity (richness and evenness of taxa within a sample) did not differ by diagnostic group, treatment, or time. In contrast, beta diversity (microbiome composition differences between samples) differed between AUD and controls and was shifted by minocycline over time. AUD, vs. controls, was associated with differences in several taxa prevalences, including reduced *Dialister* and *Subdoligranulum* and increased *Collinsella* , *Streptococcus* , and *Phascolarctobacterium* . Minocycline, vs. placebo, rapidly and robustly restructured the microbiome, altering numerous taxa, including *Lachnospira* , *Ruminococcaceae* , *Clostridium sensu stricto* , and *Christensenellaceae,* and metabolic pathways (e.g., ubiquinol biosynthesis and methylphosphonate degradation). AUD is associated with a distinct gut microbial composition, whereas minocycline produces rapid, sustained, diagnosis-independent gut microbiome restructuring, without altering alpha diversity.

